# Circulating Levels of Dephosphorylated-Uncarboxylated Matrix Gla Protein in Patients with Acute Coronary Syndrome

**DOI:** 10.3390/molecules26041108

**Published:** 2021-02-19

**Authors:** Admira Bilalic, Tina Ticinovic Kurir, Marko Kumric, Josip A. Borovac, Andrija Matetic, Daniela Supe-Domic, Josko Bozic

**Affiliations:** 1 Department of Cardiology, University Hospital of Split, Split 21000, Croatia; admirabilalic@gmail.com (A.B.); andrija.matetic@gmail.com (A.M.); 2 Department of Pathophysiology, University of Split School of Medicine, 21000 Split, Croatia; tticinov@mefst.hr (T.T.K.); kumricjudo@gmail.com (M.K.); jborovac@mefst.hr (J.A.B.); 3 Endocrinology Clinic, University Hospital of Split, 21000 Split, Croatia; 4 Institute of Emergency Medicine of Split-Dalmatia County (ZHM SDZ), 21000 Split, Croatia; 5 Department of Medical Laboratory Diagnostics, University Hospital of Split, 21000 Split, Croatia; daniela.supe.domic@ozs.unist.hr

**Keywords:** matrix Gla protein, acute coronary syndrome, vascular calcification, myocardial infarction, STEMI, NSTEMI

## Abstract

Vascular calcification contributes to the pathogenesis of coronary artery disease while matrix Gla protein (MGP) was recently identified as a potent inhibitor of vascular calcification. MGP fractions, such as dephosphorylated-uncarboxylated MGP (dp-ucMGP), lack post-translational modifications and are less efficient in vascular calcification inhibition. We sought to compare dp-ucMGP levels between patients with acute coronary syndrome (ACS), stratified by ST-elevation myocardial infarction (STEMI) and non-ST-elevation myocardial infarction (NSTEMI) status. Physical examination and clinical data, along with plasma dp-ucMGP levels, were obtained from 90 consecutive ACS patients. We observed that levels of dp-ucMGP were significantly higher in patients with NSTEMI compared to STEMI patients (1063.4 ± 518.6 vs. 742.7 ± 166.6 pmol/L, *p* < 0.001). NSTEMI status and positive family history of cardiovascular diseases were only independent predictors of the highest tertile of dp-ucMGP levels. Among those with NSTEMI, patients at a high risk of in-hospital mortality (adjudicated by GRACE score) had significantly higher levels of dp-ucMGP compared to non-high-risk patients (1417.8 ± 956.8 vs. 984.6 ± 335.0 pmol/L, *p* = 0.030). Altogether, our findings suggest that higher dp-ucMGP levels likely reflect higher calcification burden in ACS patients and might aid in the identification of NSTEMI patients at increased risk of in-hospital mortality. Furthermore, observed dp-ucMGP levels might reflect differences in atherosclerotic plaque pathobiology between patients with STEMI and NSTEMI.

## 1. Introduction

Acute coronary syndrome (ACS) represents a spectrum of clinical presentations associated with reduced myocardial blood flow [1]. Even though contemporary invasive management and antithrombotic therapies have improved mortality and morbidity outcomes associated with ACS, it remains a challenge to further mitigate the risk of in-hospital mortality, progression to heart failure, arrhythmogenesis, and future adverse events [2].

Among many other mechanisms, vascular calcification is a process of depositing calcium phosphate salts in the arterial wall and, therefore, contributes to the complex pathogenesis of coronary artery disease (CAD) [3,4]. Calcium deposits are mostly scattered in small patches throughout the intima but can also dwell in the medial layer of the blood vessel and lead to increased arterial wall stiffness and consequent reduction in arterial compliance [5,6]. The latter process is also known as Monckeberg medial sclerosis, and it is commonly found in older diabetics and patients suffering from chronic kidney disease [5,7,8].

Matrix GLA protein (MGP), a vitamin K dependent protein, on the other hand, acts as a vascular calcification inhibitor [9,10,11,12]. MGP is mainly produced by chondrocytes and vascular smooth muscle cells, whereas emerging evidence suggests that MGP is also strongly expressed in endothelial cells, a probable source of plasma-detected MGP [12,13,14,15]. The first study that demonstrated the role of MGP in inhibition of vascular calcification was undertaken by Luo et al. in 1997, showing that the knock-out mouse model with genetic deficiency of MGP (MGP −/−) developed spontaneous calcification of arteries and cartilage [16]. Afterward, several studies confirmed these findings in different animal models, whereas human studies have shown that MGP is over-expressed in the calcified atherosclerotic plaques [17,18,19,20]. 

Furthermore, data suggest that neither the un-carboxylated (ucMGP) nor dephosphorylated form of MGP (dpMGP) exerts its inhibitory function on vascular calcification properly [14,21]. Of note, since increased levels of the dephosphorylated-uncarboxylated form of MGP (dp-ucMGP) have been associated with a range of cardiovascular complications and increased mortality, we have found it important to elucidate these dynamics with respect to measuring dp-ucMGP content in plasma of patients presenting with ACS [22,23,24].

Specifically, with respect to different pathophysiological and prognostic characteristics of ACS with and without ST-segment elevation, in this study, the main goal was to compare dp-ucMGP plasma levels of consecutive patients with ST-elevation myocardial infarction (STEMI) and patients with non-ST-elevation myocardial infarction (NSTEMI) [1,25]. Furthermore, the secondary goal was to compare dp-ucMGP plasma levels with various blood parameters (electrolytes, kidney function parameters, coagulation status, troponin, C-reactive protein), family and personal history of cardiovascular diseases, use of medications, left ventricular ejection fraction and traditional cardiovascular risk factors. Finally, among patients with NSTEMI, the additional goal was to compare dp-ucMGP plasma levels between patients with and without the high risk of in-hospital mortality, defined by the Global Registry of Acute Coronary Events (GRACE) risk score > 140 points [26,27,28,29].

## 2. Results

Most of the presented baseline characteristics of patients did not differ between patients with STEMI and patients with NSTEMI (Table 1). Importantly, these two patient groups did not differ significantly with respect to age, sex distribution, body mass index, comorbidity burden and chronic pharmacotherapy use, with the exception of acetylsalicylic acid use. In comparison to the STEMI group, patients with NSTEMI had more cardiovascular-related hospitalizations (*p* = 0.001), more of them had multi-vessel disease (*p* = 0.012) and they had higher creatinine levels (*p* = 0.045). On the other hand, a greater proportion of patients with STEMI had ST segment deviation in first contact ECG (*p* = 0.002), and more performed in-hospital percutaneous coronary interventions (PCIs) (*p* = 0.001) compared to NSTEMI patients.

**Figure 1:**
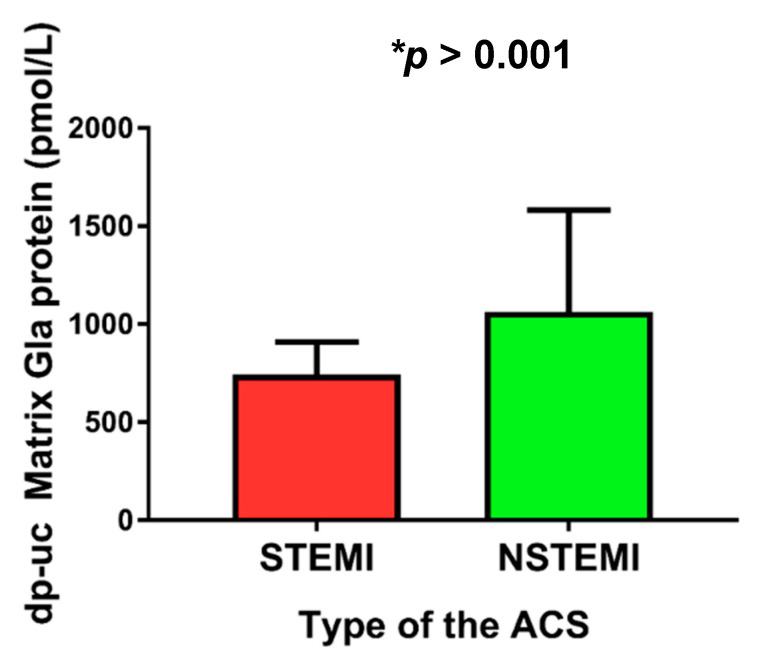
Plasma dp-uc matrix Gla protein levels with respect to different types of ACS (STEMI vs. NSTEMI). Abbreviations: dp-ucMGP: dephosphorylated and uncarboxylated matrix Gla protein; STEMI: ST-elevation myocardial infarction; NSTEMI: non-ST elevation myocardial infarction; ACS: acute coronary syndrome.

We observed that plasma levels of dp-ucMGP were significantly higher in patients with NSTEMI compared to patients with STEMI (1063.39 ± 518.58 vs. 742.74 ± 166.59 pmol/L, *p* < 0.001) (Figure 1).

**Figure 2:**
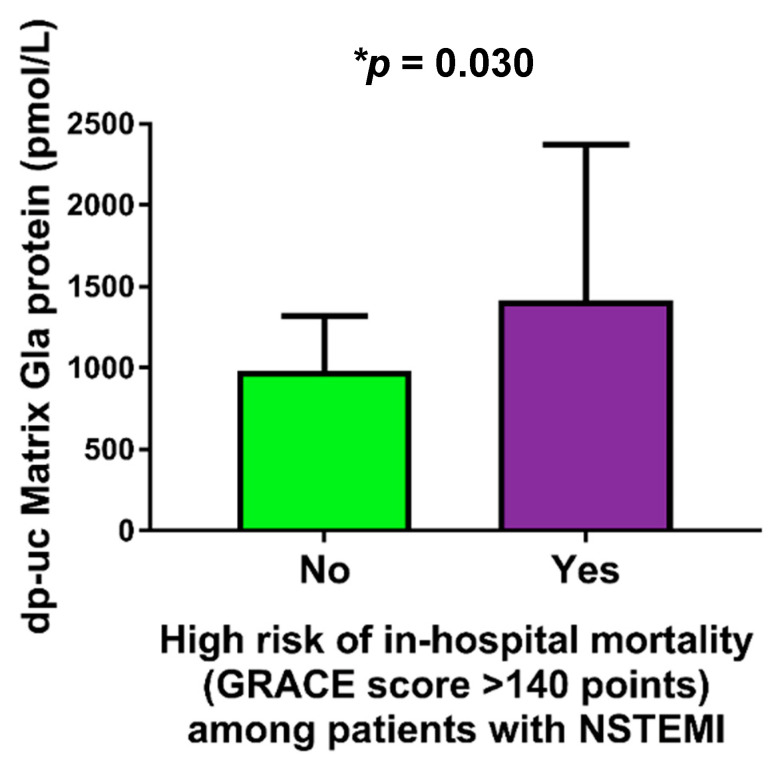
Comparison of dp-uc matrix Gla protein levels between NSTEMI patients with and without high risk of in-hospital mortality. Abbreviations: dp-ucMGP: dephosphorylated and uncarboxylated matrix Gla protein; NSTEMI: non-ST elevation myocardial infarction; GRACE: Global Registry of Acute Coronary Events; INR: international normalized ratio.

Furthermore, among patients with NSTEMI, those with high risk of in-hospital mortality (estimated by the GRACE score >140 points) had significantly higher plasma levels of dp-ucMGP in comparison to patients without high risk of in-hospital mortality (1417.75 ± 956.78 vs. 984.64 ± 335.01 pmol/L, *p* = 0.030) (Figure 2).

The univariate Pearson’s correlation analysis showed that dp-ucMGP plasma levels positively correlated with age (r = 0.259, *p* = 0.014), GRACE score (r = 0.247, *p* = 0.019) and both urea (r = 0.369, *p* < 0.001) and creatinine serum levels (r = 0.428, *p* < 0.001) in whole ACS population (Table 2). When stratified by ACS subtype, in NSTEMI population, a significant correlation was retained between dp-ucMGP and GRACE score (r = 0.312, *p* = 0.033), urea (r = 0.341, *p* = 0.024) and creatinine serum levels (r = 0.414, *p* = 0.005), while in the STEMI subgroup, the significant correlation was observed for dp-ucMGP and age (r = 0.417, *p* = 0.004) and hemostatic parameters as reflected in prothrombin time-INR (r = 0.332, *p* = 0.024) and activated partial thromboplastin time (APTT) (r = 0.432, *p* = 0.003). Other variables showed no statistically significant correlations.

**Figure 3:**
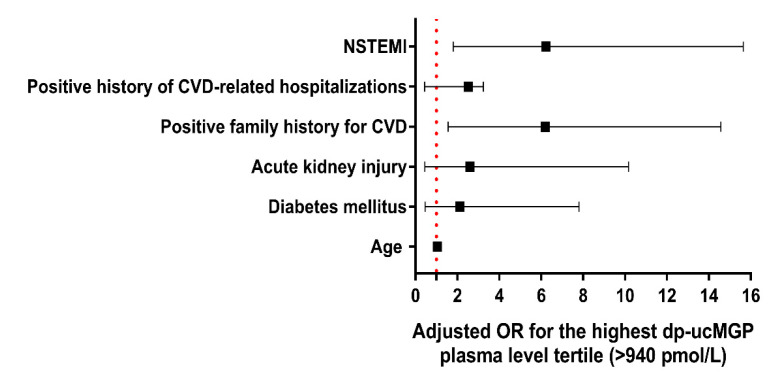
Predictors of the highest tertile of dp-ucMGP plasma levels (>940 pmol/L) after covariate adjustment in the stepwise multivariable regression model. Abbreviations: CVD: cardiovascular disease; OR: odds ratio; NSTEMI: non-ST-elevation myocardial infarction.

To ascertain which variables would be an independent predictor of the highest tertile of plasma dp-ucMGP levels (>940 pmol/L) in the whole ACS cohort, a univariate binary logistic regression was employed. This analysis showed that age, diabetes mellitus, acute kidney injury, positive family history of CVD, positive history of CVD-related hospitalizations, and NSTEMI status were single predictors of the highest tertile of plasma dp-ucMGP levels (Table 3). However, when these variables were entered into the stepwise multivariable regression model and adjusted for covariates, positive family history of CVD (OR 6.19, 95% CI 1.56–14.57) and NSTEMI as the type of ACS (6.23, 95% CI 1.80–15.64) were the only significant predictors of highest plasma dp-ucMGP levels (Figure 3). 

After adjustment for confounding variables in the model of stepwise multivariable logistic regression, the dp-ucMGP plasma level as a continuous variable was significantly associated with positive NSTEMI status (OR 1.004, 95% CI 1.001–1.006) (Table 4).

## 3. Discussion

The present cross-sectional study provided several important results. One of the main findings is that the dp-ucMGP plasma levels were significantly higher among ACS patients with NSTEMI in comparison to patients with STEMI. In addition, after adjustment for confounding variables in the model of multivariable logistic regression, circulating dp-ucMGP was associated with positive NSTEMI status while, on the other hand, NSTEMI status and positive family history for CVD were the only independent predictors of the highest tertile of plasma dp-ucMGP levels in the multivariable-adjusted regression model performed across the whole cohort of patients. When further refined, it is those patients with NSTEMI at the highest risk of in-hospital mortality (estimated by the GRACE score) who had significantly higher plasma levels of dp-ucMGP compared to NSTEMI patients in the lower-risk categories. In addition, GRACE score values positively correlated with dp-ucMGP levels. Apart from the main findings, dp-ucMPG plasma levels have been shown to positively correlate with urea and creatinine levels, and this relationship was pronounced in NSTEMI but not STEMI patients. To the best of our knowledge, this is the first original study to compare the dp-ucMGP levels between patients with different subtypes of ACS.

The observed difference in the dp-ucMGP plasma levels could likely be explained by differences in atherosclerotic plaque pathobiology between patients with STEMI and NSTEMI. In a recent study, Dong et al. demonstrated that patients with NSTEMI had a higher prevalence of calcified thick-cap fibroatheromas and larger superficial calcium percentage at the minimum lumen cross-sectional area (MLA) than patients with STEMI [20]. Intravascular imaging study showed more hyperechoic and less of hypoechoic and lipid-pool-like image lesions in NSTEMI vs. STEMI patients [30]. In addition, patients with NSTEMI had a more pronounced culprit lesion calcium arc as opposed to the STEMI counterparts [30]. Since earlier studies have established a correlation between the dp-ucMGP plasma levels and the extent of vascular calcification in various pathologic states, it is plausible that this could, at least in part, explain the observed difference [31,32,33,34,35]. Taken together, it is likely that higher circulating dp-ucMGP levels might reflect more calcified coronary lesions and higher vascular calcification burden. In addition to this notion, we observed that age was a significant univariate predictor of the highest dp-ucMGP levels; however, this significance was lost in the multivariable model. This is of potential relevance since it is well-established that vascular calcification is highly dependent on age while older patients tend to present with highly calcified and tortuous coronary lesions compared to their younger counterparts [36]. It is also noteworthy that our study had significantly more NSTEMI patients with multi-vessel disease in contrast to patients with STEMI. This could also to some extent explain the detected difference in dp-ucMGP concentrations between the two groups, although multivessel disease was not found as a significant predictor of high dp-ucMGP levels in our study. However, the observed relationship could likely be due to the relatively insufficient sample size and we hold that this relationship is worthy of further investigation in larger patient cohorts.

Furthermore, in our study, we measured significantly higher dp-ucMGP plasma levels among NSTEMI patients at higher risk of in-hospital mortality (as assessed by the GRACE score, which holds IIa recommendation in current ESC NSTEMI guidelines for the purposes of risk stratification) [28]. This finding indicates a possible role of the dp-ucMGP in NSTEMI mortality prediction. Earlier studies reported conflicting data regarding the association between elevated dp-ucMGP plasma levels and poor outcomes in some CV diseases. Specifically, Mayer et al. reported that circulating dp-ucMGP was independently associated with the risk of all-cause and cardiovascular mortality, with the dp-ucMGP being the strongest predictor of mortality in subjects with lower cardiovascular risk [37]. Additional studies demonstrated the same in diabetics and highlighted that the risk assessment with dp-ucMGP is independent of the classical risk factors and vitamin D status [38,39]. Conversely, in a prospective case-cohort study, Dalmeijer et al. reported that the dp-ucMGP levels were not associated with increased CAD risk, whereas Mendelian randomization study in a Flemish population showed that the higher dp-ucMGP levels predict total, non-cancer and cardiovascular mortality, but lower coronary risk [40,41]. In our study, all included patients survived to discharge, therefore, it was not possible to undertake analysis of dp-ucMGP levels with respect to in-hospital mortality. Of note, as discussed by Wang et al., coronary artery calcium (CAC) scores improve cardiovascular risk discrimination and reclassify a proportion of intermediate risk individuals [42]. As dp-ucMGP can reflect early signs of vascular calcification, Vassalle et al. argue that plasma dp-ucMGP could be used in CVD risk assessment instead of CAC [43]. Notwithstanding, each novel biomarker has to be assessed by its appropriateness to answer fundamental questions in order to judge its clinical relevance: whether the biomarker provides additional information beyond traditional biomarkers, to which group of patients should the marker be applied, and at which point in time should the marker be measured.

Finally, a positive correlation of circulating dp-ucMGP levels with established kidney status markers (urea and creatinine) that we observed is in line with conducted studies. In our study, this relationship was more pronounced among NSTEMI compared to STEMI patients. Schurgers et al. demonstrated that plasma dp-ucMGP levels augmented progressively with chronic kidney disease stage and in a recent study by Fain et al., authors reported higher dp-ucMGP levels in patients on hemodialysis [44,45]. Of note, acute kidney injury was also a predictor of highest tertile of dp-ucMGP levels in the whole cohort of ACS patients; however, this significance was not retained after adjustment for other variables in the model. Among the parameters that we did not compare with dp-ucMGP in our study, and which should be addressed in further studies, Lipoprotein(a) (Lp(a)) is important to point out. Apart from being an established cardiovascular risk factor, Lp(a) recently emerged as a potential biomarker for CAC volume progression [46]. Moreover, earlier studies demonstrated positive correlation between Lp(a) and MGP levels hypothesizing that the high levels of MGP associated with Lp(a) are owing to a negative feedback loop [47,48]. In addition, Lp(a) also emerged as a viable therapeutic target since it has been shown that it may significantly contribute to residual cardiovascular risk in patients with CAD and optimal LDL-C levels [49]. Of important note, we have not included healthy controls in our study, yet data from available studies that included healthy controls similar to our population implicate that healthy controls have significantly lower plasma levels of dp-ucMGP in comparison to either patients with STEMI or NSTEMI [15,50,51].

The present study has several limitations. First, and the main limitation, is that the study is of cross-sectional design, which prevents us from making any causal inferences. Secondly, we performed only a single measurement of dp-ucMGP levels during the admission and evaluation of patients with ACS; therefore, we lack information on possible dp-ucMGP dynamics. Thirdly, given the complex immunopathological and pathophysiological development of ACS in temporal aspect and with respect to myocardial injury and plaque burden, it is likely that blood samples were taken heterogeneously at different time points and periods of admission of ACS patients; thus, the obtained dp-ucMGP levels might not uniformly reflect disease state. Furthermore, a group of healthy controls was not included in the present study. Finally, our study lacks mortality/morbidity outcomes, it was conducted in a single center and it included a relatively small number of participants.

## 4. Materials and Methods

### 4.1. Study Design and Ethical Considerations

This cross-sectional study was performed at the University Hospital of Split during the period from June 2018 to July 2019.

The study was approved by the Ethics Committee of University Hospital of Split and was conducted in accordance with all ethical principles of the Helsinki Declaration from 2013. Before enrolling in the study, every participant was informed about the procedures, course and purpose of this research and each of them individually signed an informed written consent.

### 4.2. Subjects and Inclusion/Exclusion Criteria

The present study included 90 consecutive patients ≥18 years of age diagnosed with ACS, 46 of which were patients with STEMI and 44 were patients with NSTEMI. Patients were diagnosed and treated in accordance with European Society of Cardiology (ESC) guidelines for treatment of STEMI and NSTEMI [27,28]. Exclusion criteria were: state of circulatory shock at the time of admission (defined by current ESC guidelines), active malignancy (irrelevant of the stage or type of malignancy), disorders of bone metabolism (rickets, osteomalacia, osteogenesis imperfecta, osteopetrosis fibrous dysplasia and Paget disease) or medications/supplements that significantly alter bone metabolism (corticosteroids, bisphosphonates, cyclosporine), premenopausal state in women subjects.

### 4.3. Clinical and Laboratory Evaluations

For the body weight (kg) and height (cm) measurements, we used a calibrated scale (Seca, Birmingham, UK) and the BMI was calculated by the body weight (kg) being divided by height-squared (m2 waist circumference (cm) was measured while standing at the mid-point between the inferior tip of the ribcage and the superior aspect of the iliac crest, while hip circumference (cm) was measured at the point providing maximum circumference over the buttocks, using a tape measure. Waist-to-hip ratio (WHR) was calculated by dividing the waist by the hip circumference.

Physical examination and medical history items were directly collected from all patients included in the study in the first 24 h of admission. For the body weight (kg) and height (cm) measurements, we used a calibrated scale (Seca, Birmingham, UK) and the BMI was calculated by the body weight (kg) being divided by height-squared (m2 waist circumference (cm) was measured while standing at the mid-point between the inferior tip of the ribcage and the superior aspect of the iliac crest, while hip circumference (cm) was measured at the point providing maximum circumference over the buttocks, using a tape measure. WHR was calculated by dividing the waist by the hip circumference. A transthoracic echocardiography (TTE) examination was performed in the first 24 h of admission. All measurements were taken while patients were at rest and in the left lateral decubitus position; LV ejection fraction (LVEF) was measured several times by the 2D biplane method, according to the modified Simpson’s rule, and the average value was recorded. All images were acquired using the Vivid 9 ultrasound system (GE Medical Systems, Milwaukee, WI, USA) and stored/analyzed on the Echo PAC workstation (EchoPac PC, version 112; GE Medical Systems, Milwaukee, WI, USA). The severity of heart failure and left ventricular dysfunction was clinically assessed by using Killip classification, while in-hospital mortality risk was assessed by the GRACE score [26,29]. The GRACE score is a scoring system used to stratify ACS patients with respect to their in-hospital and 6-month to 3-year mortality [26]. Current ESC guidelines stipulate an early invasive strategy within 24 h in patients with NSTEMI and GRACE scores >140 points, while the GRACE score is endorsed with class recommendation IIa and level of evidence B for prognosis estimation in NSTEMI [28]. Similarly, the GRACE risk score is recommended for risk assessment and treatment adjustment in STEMI also; however, no formal level of recommendation is supported in the latest STEMI guidelines with respect to usage of this score [27]. Peripheral blood samples were collected in test tubes with anticoagulant on admission and were afterwards centrifuged and stored at −80 °C for further analysis. Blood samples were analyzed by an identical specialist in medical biochemistry, who was blind to the subject group in the study. Plasma dp-ucMGP levels were analyzed by chemiluminescent immunoassay (CLIA) method using IDS-iSYS InaKtif MGP (Immunodiagnostic Systems, Frankfurt, Germany) according to the manufacturer’s instructions. We performed a paired measurement based on which we determined the mean value. The minimum limit of detection was 200 pmol/L. Inter-assay and intra-assay coefficients of variability (CV) were 7.9% and 4.5%, respectively. Hs-cTnI was determined by chemiluminescent microparticle immunoassay (CMIA) using ARCHITECT STAT High Sensitive Troponin-I assay (Abbott Laboratories, Illinois, United States) according to the manufacturer’s instructions. Other laboratory parameters were measured according to standard laboratory procedures.

### 4.4. Statistical Analysis

Data were analyzed by using SPSS Statistics for Windows^®^ (version 26.0, IBM, Armonk, NY, USA) and Prism 6 for Windows^®^ (version 6.01, GraphPad, La Jolla, CA, USA). Categorical data were shown as absolute numbers (N) and percentages (%), while continuous data were shown as mean ± standard deviation (SD) or median (interquartile range). The normality of data was assessed with the Kolmogorov–Smirnov test. Differences between two principal groups of interest (STEMI vs. NSTEMI cohort) were assessed by using independent samples t-test and Mann–Whitney U test for continuous variables and Chi-squared (χ^2^) test for categorical variables or Fisher’s exact test, where appropriate. For the association of plasma dp-ucMGP levels with continuous baseline anthropometric, clinical, and laboratory parameters, a Pearson’s bivariate correlation analysis was used. In this analysis, the respective r correlation coefficient (rho) and two-tailed significance (*p*) values were reported. To determine which clinical variables would be independent predictors of the highest tertile of plasma dp-ucMGP levels (>940 pmol/L) in the whole ACS cohort, a univariate binary logistic regression was employed. This analysis evaluated relevant clinical variables such as age, sex, BMI, diabetes mellitus, arterial hypertension, dyslipidemia, acute kidney injury, positive family history for cardiovascular disease (CVD), positive history of CVD-related hospitalizations, left-ventricular ejection fraction (LVEF), multivessel disease and NSTEMI status as single predictors of highest tertile of plasma dp-ucMGP levels. Variables significant (*p* < 0.05) in the univariate regression model were entered in the stepwise multivariable regression model. Finally, to determine if the dp-ucMGP levels were independently associated with positive NSTEMI status, the stepwise multivariable logistic regression analysis was used and was adjusted for potential confounding variables at baselines such as age, sex, body mass index (BMI), systolic blood pressure, renal function (serum creatinine), C-reactive protein, left ventricular ejection fraction (LVEF), and relevant comorbidities encompassing arterial hypertension, type II diabetes mellitus, dyslipidemia and smoking. For this analysis, respective odds ratios (ORs), 95% confidence intervals (CIs), and two-tailed *p*-values were reported. Results with *p*-value <0.05 were considered statistically significant in all analyses.

## 5. Conclusions

In this study, for the first time, we demonstrated that the dp-ucMGP plasma levels are higher in ACS patients with NSTEMI in comparison to STEMI. Furthermore, among patients with NSTEMI, higher plasma levels of dp-ucMGP were associated with a greater risk of in-hospital mortality, which is consistent with the previous data that suggest that high dp-ucMGP levels reflect mortality rates in CVDs. Nevertheless, these findings should be supported with additional evidence in future, well-designed studies.

## Figures and Tables

**Table 1:** Baseline characteristics of patients.

Variable	Overall (*n* = 90)	STEMI (*n* = 46)	NSTEMI (*n* = 44)	*p*-Value
Age, years	67.2 ± 9.1	66.2 ± 8.8	68.2 ± 9.4	0.308 ^1^
Male sex	71 (78.9%)	35 (76.1%)	36 (81.8%)	0.505 ^1^
Body mass index, kg/m^2^	27.08 ± 2.54	27.45 ± 2.71	26.70 ± 2.31	0.158 ^3^
Waist-to-hip ratio	1.04 ± 0.68	1.04 ± 0.68	1.04 ± 0.69	0.630 ^3^
Systolic blood pressure, mmHg	134.7 ± 21.0	134.3 ± 21.3	135.1 ± 20.9	0.845 ^3^
Diastolic blood pressure, mmHg	79.7 ± 12.6	81.2 ± 12.3	78.1 ± 12.9	0.252 ^3^
Heart rate at admission, bpm	75.0 ± 16.9	76.7 ± 18.6	73.3 ± 14.9	0.353 ^3^
Diabetes mellitus	13 (14.4%)	4 (8.7%)	9 (20.5%)	0.140 ^2^
Arterial hypertension	51 (56.7%)	27 (58.7%)	24 (54.5%)	0.832 ^1^
Smoking	43 (47.8%)	24 (52.2%)	19 (43.2%)	0.393 ^1^
Dyslipidemia	14 (15.6%)	6 (13.0%)	8 (18.2%)	0.501 ^1^
Atrial fibrillation	12 (13.3%)	8 (17.4%)	4 (9.1%)	0.355 ^2^
Family history of cardiovascular disease	17 (18.9%)	10 (21.7%)	7 (15.9%)	0.480 ^1^
History of PCI or CABG	16 (17.8%)	4 (8.7%)	12 (27.3%)	0.028 ^2^
History of CV-related hospitalizations	24 (26.7%)	5 (10.9%)	19 (43.2%)	0.001 ^2^
GRACE score, points	121.4 ± 22.0	121.1 ± 23.1	121.7 ± 23.1	0.906 ^3^
Left ventricular ejection fraction, %	50.7 ± 10.3	51.2 ± 9.7	50.1 ± 11.1	0.652 ^3^
Mean Killip class *	1.0 (1.0-1.0)	1.0 (1.0-1.0)	1.1 (1.0-1.0)	0.136 ^4^
ST segment deviation in first contact ECG	82 (91.1%)	46 (100%)	36 (81.8%)	0.002 ^1^
PCI performed while in-hospital	74 (82.2%)	44 (95.7%)	30 (68.2%)	0.001 ^1^
Multi-vessel disease	10 (13.3%)	2 (4.4%)	8 (26.7%)	0.012 ^2^
Beta-blocker use	29 (32.2%)	12 (26.1%)	17 (38.6%)	0.203 ^1^
ACE inhibitor or ARB use	41 (45.6%)	21 (45.7%)	20 (45.5%)	0.985 ^1^
Calcium channel blocker use	14 (15.6%)	8 (17.4%)	6 (13.6%)	0.623 ^1^
Statin use	18 (20.0%)	6 (13.0%)	12 (27.3%)	0.092 ^1^
Diuretic use	20 (22.2%)	8 (17.4%)	12 (27.3%)	0.260 ^1^
Acetylsalicylic acid use	23 (25.6%)	7 (15.2%)	16 (36.4%)	0.021 ^1^
P_2_Y_12_ inhibitor use	4 (4.4%)	1 (2.2%)	3 (6.8%)	0.355 ^2^
Anticoagulant use	7 (7.8%)	4 (8.7%)	3 (6.8%)	1.000 ^2^
Prothrombin time—INR	1.09 ± 0.39	1.07 ± 0.25	1.11 ± 0.50	0.608 ^3^
Activated partial thromboplastin time, s	24.13 ± 4.02	23.96 ± 4.21	24.32 ± 3.85	0.672 ^3^
C-reactive protein, mg/L	6.4 ± 5.0	6.6 ± 5.2	6.2 ± 4.9	0.664 ^3^
High-sensitivity cardiac troponin I at admission, ng/L	331.0 ± 293.8	335.2 ± 306.1	326.9 ± 284.8	0.894 ^3^
Potassium, mmol/L	4.04 ± 0.40	3.98 ± 0.39	4.10 ± 0.41	0.176 ^3^
Urea, mmol/L	7.6 ± 3.2	7.0 ± 2.2	8.2 ± 4.0	0.094 ^3^
Creatinine, μmol/L	0.428	88.0 ± 21.6	102.9 ± 44.3	0.045 ^3^

Data are presented as mean ± standard deviation or n (%); * Data presented as median (interquartile range); ^1^ Chi-squared test; ^2^ Fisher’s exact test; ^3^ t-test for independent samples; ^4^ Mann–Whitney U test; WHO: World Health Organization; COPD: chronic obstructive pulmonary disease; PCI: percutaneous coronary intervention; CABG: coronary artery bypass grafting, CV: cardiovascular; ACE: angiotensin converting enzyme; ARB: angiotensin receptor blocker; INR: international normalized ratio.

**Table 2:** Significant univariate Pearson’s correlations between dp-ucMGP plasma levels and anthropometric, clinical and laboratory variables.

Variable	r-Correlation Coefficient	*p*-Value ^1^
Age, years	0.259	0.014
GRACE score, points	0.247	0.019
Prothrombin time—INR	0.257	0.015
Activated partial thromboplastin time, s	0.251	0.017
Second high-sensitivity cardiac troponin I, ng/L	0.236	0.026
Urea, mmol/L	0.369	<0.001
Creatinine, μmol/L	0.428	<0.001

^1^ Univariate Pearson’s correlation. Abbreviations: GRACE: Global Registry of Acute Coronary Events; INR: international normalized ratio.

**Table 3:** Predictors of highest tertile of plasma dp-ucMGP levels in the total ACS patient cohort (*n* = 90), derived from binary logistic regression analysis.

Variable	Univariate Model			Multivariate Model		
	OR	95% CI	*p*-Value	OR	95% CI	*p*-Value ^1^
Age	1.06	1.01–1.12	0.034	1.04	0.98–1.11	0.220
Sex	1.62	0.57–4.59	0.364	-	-	-
BMI	1.09	0.91 –1.29	0.346	-	-	-
Diabetes mellitus	4.00	1.18–13.57	0.026	2.12	0.46–7.80	0.336
Arterial hypertension	1.87	0.75–4.66	0.178	-	-	-
Dyslipidemia	2.30	0.73–7.32	0.157	-	-	-
Acute kidney injury	4.75	1.10–20.57	0.037	2.60	0.45–10.16	0.289
Positive family history for CVD	3.79	1.27–11.31	0.017	6.19	1.56–14.57	0.010^1^
Positive history of CVD-related hospitalizations	3.27	1.17–9.16	0.024	2.53	0.44–3.24	0.849
NSTEMI	4.75	1.81–12.46	0.002	6.23	1.80–15.64	0.004 ^1^
Multivessel disease	3.06	0.78–11.96	0.107	-	-	-
LVEF	0.98	0.94–1.04	0.631	-	-	-
High GRACE score (>140 points)	1.36	0.47–3.95	0.577	-	-	-

^1^ Statistical significance determined at the two-tailed level in multivariate-adjusted stepwise regression model including covariates of age, diabetes mellitus, acute kidney injury, positive family history for CVD, positive history of CVD-related hospitalizations, and NSTEMI as the type of ACS. Abbreviations: BMI: body mass index; CI: confidence interval; CVD: cardiovascular disease; GRACE: Global Registry of Acute Coronary Events; LVEF: left-ventricular ejection fraction; NSTEMI: non-ST-elevation myocardial infarction; OR: odds ratio.

**Table 4:** Association of dp-ucMGP plasma levels with NSTEMI status adjusted for other confounding variables in the model of stepwise multivariable logistic regression.

Variable	Odds Ratio	95% CI	*p*-Value
dp-ucMGP, pmol/L	1.004	1.001–1.006	0.011 ^1^
Age, years	1.018	0.948–1.092	0.629
Sex	0.626	0.147–2.654	0.525
Body mass index, kg/m^2^	0.856	0.692–1.059	0.153
Creatinine, μmol/L	0.999	0.974–1.024	0.917
C-reactive protein, mg/L	0.996	0.980–1.011	0.580
Left-ventricular ejection fraction, %	0.993	0.938–1.050	0.795
Systolic blood pressure, mmHg	1.007	0.980–1.036	0.600
Smoking	0.987	0.294–3.315	0.983
Diabetes mellitus type II	0.970	0.161–5.852	0.973
Arterial hypertension	0.557	0.182–1.704	0.305
Dyslipidemia	2.439	0.486–12.227	0.278

^1^ Multivariable logistic regression. Abbreviations: dp-ucMGP: dephosphorylated-uncarboxylated matrix Gla protein.

## Data Availability

The data presented in this study are available on request from the corresponding author. The data are not publicly available because some of the data set will be used for further research.

## Sample Availability

Samples of the compounds are not available from the authors.
